# Optimizing Propagation of *Staphylococcus aureus* Infecting Bacteriophage vB_SauM-phiIPLA-RODI on *Staphylococcus xylosus* Using Response Surface Methodology

**DOI:** 10.3390/v10040153

**Published:** 2018-03-27

**Authors:** Eva González-Menéndez, Francisco Noé Arroyo-López, Beatriz Martínez, Pilar García, Antonio Garrido-Fernández, Ana Rodríguez

**Affiliations:** 1 Instituto de Productos Lácteos de Asturias (IPLA-CSIC), Paseo Río Linares s/n, 33300 Villaviciosa, Spain; eva.gm@ipla.csic.es (E.G.-M.); bmf1@ipla.csic.es (B.M.); pgarcia@ipla.csic.es (P.G.); 2 Departamento de Biotecnología de Alimentos, Instituto de la Grasa (IG-CSIC), Campus Universitario Pablo de Olavide, Edificio 46. Ctra, Sevilla-Utrera, 1 km, 41013 Sevilla, Spain; fnoe@ig.csic.es (F.N.A.-L.); garfer@cica.es (A.G.-F.)

**Keywords:** bacteriophages, food safety, propagation, optimization, Response Surface Methodology, *Staphylococcus*

## Abstract

The use of bacteriophages for killing pathogenic bacteria is a feasible alternative to antibiotics and disinfectants. To obtain the large quantities of phages required for this application, large-scale production of bacteriophages must be optimized. This study aims to define conditions that maximize the phage yield of the virulent and polyvalent staphylococcal bacteriophage vB_SauM-phiIPLA-RODI in broth culture, using the food-grade species *Staphylococcus xylosus* as the host strain to reduce the risk of growing massive quantities of pathogenic bacteria and therefore, to ensure the safety of the final phage stock. The effect of four variables, namely initial bacterial concentration (5.66–8.40 log_10_ colony-forming unit (CFU)/mL), initial phage concentration (5–8 log_10_ plaque-forming unit (PFU)/mL), temperature (21–40 °C) and agitation (20–250 rpm), on phage yield (response) was studied by using response surface methodology (RSM). Successive experimental designs showed that agitation did not significantly impact phage yield, while temperature did have a significant effect, with 38 °C being the optimum for phage propagation. The results allowed the design of a model to describe phage yield as a function of the initial bacterial and phage concentrations at fixed agitation (135 rpm), and optimum temperature (38 °C). The maximum experimental phage yield obtained was 9.3 log_10_ PFU/mL, while that predicted by the model under the optimized conditions (7.07 log_10_ CFU/mL initial bacterial population and 6.00 log_10_ PFU/mL initial phage titer) was 9.25 ± 0.30 log_10_ PFU/mL, with the desirability of 0.96. This yield is comparable to that obtained when the phage was propagated on the original host, *Staphylococcus aureus*. Bacteriophage phiIPLA-RODI showed the same host range and very similar biofilm removal ability regardless of the staphylococcal species used for its propagation. The results presented in this study show the suitability of using a food-grade strain of *S. xylosus* for the propagation of *S. aureus* infecting phages and the application of RSM to define the optimal propagation conditions.

## 1. Introduction

Staphylococcal food poisoning is one of the most common food-borne diseases worldwide that results from the ingestion of pre-formed enterotoxins produced by *Staphylococcus aureus* [1]. The European Food Safety Authority (EFSA) reported in 2015 a total of 434 food-borne outbreaks caused by staphylococcal toxins [2]. In the United States, 352 outbreak-associated illnesses and 27 hospitalizations were caused by *S. aureus* toxins in 2016 [3]. The pathogenicity of this bacterium is due to a combination of toxin-mediated virulence, invasiveness and antibiotic resistance [4]. Indeed, methicillin-resistant *S. aureus* (MRSA) is one of the leading causes of hospital-acquired infections.

It is also remarkable the increase in infections caused by multidrug-resistant *S. aureus* and community-associated methicillin-resistant *S. aureus* (CA-MRSA) as it results in a diminished effectiveness of the antibiotic treatment [5]. For instance, many methicillin-resistant strains of *S. aureus* (MRSA) show a decreased susceptibility to glycopeptides such as vancomycin [6,7,8]. An interesting survey addressed by the European Centre for Disease Prevention and Control (ECDC) in the period 2011–2012 showed a 41% of invasive *S. aureus* isolates carrying methicillin resistance [9].

Since multidrug resistance is rapidly evolving in several species including *S. aureus* [10], there is a clear need for novel approaches to circumvent this problem. Bacteriophages have been proposed as a suitable antimicrobial alternative to the use of antibiotics, as they are natural enemies of bacteria [11,12]. Currently, there is a renaissance of phage therapy studies in western countries, and the results from clinical trials in animals are confirming the efficacy of phages as therapeutics. Despite the regulatory hurdles, several biotechnological companies are assaying bacteriophages in human clinical trials to promote their future commercialization (reviewed by [13]). Bacteriophages are also suitable to control pathogenic bacteria along the food chain [14]. Several studies have demonstrated their effectiveness in controlling bacterial pathogens in agro-food industry, such as *Salmonella*, *S. aureus*, *Campylobacter jejuni*, *Escherichia coli* O157: H7, and *Listeria monocytogenes* [15]. Usefulness of bacteriophages in food safety includes their application as disinfectants to remove bacterial biofilms from industrial surfaces [16,17,18] and the development of tools to improve pathogen detection [19]. Since the approval of phage-based products to use in the food industry sector by the US FDA in 2006, some new bacteriophage products are commercially available. These products consist of a mixture of one or more bacteriophages infecting *L. monocytogenes* (PhageGuard Listex, Micreos Food Safety B.V., Wageningen, The Netherlands; ListShieldTM, Intralytix Inc., Baltimore, MD, USA), *E. coli* O157: H7 (EcoShieldTM, Intralytix Inc., Baltimore, MD, USA) and *Salmonella* (SalmoFreshTM, Intralytix Inc., Baltimore, MD, USA; PhageGuard S, Micreos Food Safety B.V., Wageningen, The Netherlands).

We had previously isolated and characterized the *S. aureus* infecting phage vB_SauM-phiIPLA-RODI (in short, phiIPLA-RODI) that belongs to the *Myoviridae* family and exhibits a wide host range [16]. In challenge assays against *S. aureus* cultures, the phage reduced the viable counts by 5 log units in 8 h. Moreover, exposure of biofilms to this phage also reduced adhered bacteria by 2 log units [16]. Therefore, the lytic ability of phiIPLA-RODI against planktonic and sessile cells supports its potential as an antimicrobial to both remove staphylococcal biofilms and to treat *S. aureus* infections.

The use of phages as antimicrobials in the clinic and the food industry requires large-scale and reproducible production of phage cultivation. Temperature, media composition, bacteria and bacteriophage concentration are factors that can affect phage production [20].

In this regard, the aim of the present work was to optimize the propagation of phage phiIPLA-RODI by using a food-grade species instead of the original pathogenic host in a laboratory scale applying the response surface methodology (RSM). This methodology is a collection of mathematical and statistical techniques that allow the analysis of the relationship between a set of controllable experimental factors and the observed results of the variable of interest (response) to optimize the response [21,22]. Unlike the present work, the scarce studies on the production of bacteriophages that have already applied RSM have used the original bacterial host and other factors that affect phage production [23].

We used a food-grade strain of the species *Staphylococcus xylosus* as an alternative host to reduce the risk of growing massive quantities of a bacterial pathogen. The effects of different levels of the expected influential variables such as temperature, agitation, initial bacterial and bacteriophage concentration were investigated by using RSM. The equation at the region of maximum phage yield was validated, and a definitive model incorporating the validation data was deduced. Based on this model, and using the desirability approach, the optimal operating conditions were established.

## 2. Materials and Methods

### 2.1. Bacterial Strains, Bacteriophage and Media

Staphylococcal strains were isolated in Baird-Parker (BP) agar supplemented with egg yolk tellurite emulsion (Scharlau, Barcelona, Spain) and routinely grown in Tryptic Soy Broth (TSB, Scharlau, Barcelona, Spain) with shaking (Excella E24 Incubator Shaker, New Brunswich Scientific, Edison, NJ, USA) or Tryptic Soy Agar (TSA), at 37 °C.

The food-grade strain *Staphylococcus xylosus* CTC1642, isolated from a fermented meat product (IRTA, Monells, Girona, Spain) and the strain *S. aureus* IPLA1, isolated from a dairy product (IPLA-CSIC, Villaviciosa, Asturias, Spain), were used to propagate the *S. aureus*-infecting bacteriophage phiIPLA-RODI.

Phage titer was assessed by plaque assay. One-hundred μL of an overnight culture (about 10^9^ colony-forming unit (CFU)/mL) of the host strains were mixed with 100 μL of serial phage dilutions. These mixtures were added to 5 mL of molten TSA overlay (0.7% agar), poured onto TSA plates, incubated at 37 °C for 18–24 h, and the lysis plaques counted [16].

For selecting the food-grade host for phage propagation purposes, preliminary plaque assays with phage phiIPLA-RODI were performed on several food-grade strains, kindly provided by Dr Margarita Garriga (IRTA, Monells, Girona, Spain): *S. xylosus* strains (CTC1638, CTC1642 and CTC1644) and *Staphylococcus carnosus* strains (CTC6064, CTC6071 and CTC6072). The strain *S. carnosus* TM300, kindly provided by Dr Gabi Bierbaum (University of Bonn, Germany) was also tested. The efficiency of plating (EOP) was calculated by dividing the phage titer obtained on each of the tested strains by the phage titer on the reference strain *S. aureus* IPLA1.

The host range of phiIPLA-RODI lysates obtained by propagation on CTC1642 and IPLA1 was determined by plaque assay on some other staphylococcal species previously tested [16]. Some of them such as *S. lugdunensis* ZL5-11, *S. pasteuri* ZL16-6, *S. arlettae* ZL114-5, *S. xylosus* ZL61-2, *S. gallinarum* ZL90-5 and *S. kloosii* ZL74-2, were isolated from women’s breast milk [16], while others (*S. aureus* IPLA15 and IPLA16) were isolated from meat industry surfaces [24]. The efficiency of plating (EOP) of both phage lysates on each strain was calculated as indicated above.

### 2.2. Biofilm Removal by Phage phiIPLA-RODI Propagated on S. xylosus CTC1642 and S. aureus IPLA1

Overnight cultures of *S. aureus* IPLA16 were diluted to 10^6^ CFU/mL into fresh TSB supplemented with 0.25% glucose. Aliquots of 200 μL of each culture were poured into the wells of a polystyrene microtiter plate (TC Microwell 96U w/lid nunclon DSI plates, Thermo Scientific, Madrid, Spain). Biofilms were grown for 24 h at 37 °C. Wells were then washed twice with PBS buffer (137 mM NaCl, 2.7 mM KCl, 10 mM 138 Na_2_HPO_4_ and 2 mM KH_2_PO_4_; pH 7.4). To compare the biofilm degradation ability of each phage lysate, 200 μL of phiIPLA-RODI propagated on *S. aureus* IPLA1 or *S. xylosus* CTC1642 were added to each well (10^8^ plaque-forming unit (PFU)/well). SM buffer (20 mM Tris HCl, 10 mM of MgSO_4_, 10 mM of Ca(NO_3_)_2_ and 0.1 M of NaCl, pH 7.5) was added for control purposes. The microwell plates were incubated for 4 h at 37 °C. The supernatants were removed, and wells washed once with SM buffer (20 mM Tris HCl, 10 mM MgSO_4_, 10 mM Ca(NO_3_)_2_ and 0.1 M NaCl, pH 7.5) and air-dried for 15 min at room temperature. The biomass adhered to the wells was determined by crystal violet (0.1%, *w*/*v*) staining as described previously [25]. All the assays were performed using three biological replicates.

### 2.3. One-Step Growth Curve

One-step growth curve assays were carried out with phage phiIPLA-RODI, using the sensitive strains *S. aureus* IPLA1 and *S. xylosus* CTC1642. A standardised protocol, previously described [26], was adapted for this study.

Curves were performed in TSB supplemented with Ca(NO_3_)_2_ (10 mmol L^−1^) and MgSO_4_ (10 mmol L^−1^) using a multiplicity of infection (MOI) of 0.01. Mid-exponential-phase cultures (10 mL) of *S. aureus* IPLA1 and *S. xylosus* CTC1642 (OD_600_ = 0.1) were collected by centrifugation and suspended into 1 mL of fresh TSB. The phage was added and allowed to adsorb for 5 min at 37 °C with shaking. The mixture was then centrifuged, pelleted cells resuspended in 10 mL of TSB, and incubation continued at 37 °C. Samples were first taken at 5 min intervals for 30 min, and subsequently at 10 min intervals. Each sample was immediately diluted and plated for phage titration.

### 2.4. Bacteriophage Amplification: Conventional Phage Propagation

Bacteriophage phiIPLA-RODI was routinely propagated on *S. aureus* IPLA1 and *S. xylosus* CTC1642, according to the following procedure: TSB broth was inoculated with 1% (*v*/*v*) inoculum of an overnight culture of the strains indicated above, and incubated at 37 °C with shaking until an OD_600_ = 0.1 (10^7^ CFU/mL) was reached. Phage was added to the bacterial culture at MOI of 1.0 and incubation proceeded for a further 3.5 h at 37 °C with shaking. Phage preparations were obtained by centrifugation and further filtration to remove bacterial cells and debris.

The phage titer was determined by the plaque assay using 100 µL of an *S. aureus* IPLA1 overnight culture as a host, and 100 µL of the phage dilution. This mixture was added to 5 mL of molten TSA overlay (0.7% agar) and poured onto TSA plates and incubated at 37 °C for 18–24 h [16].

### 2.5. Bacteriophage Amplification: Phage Propagation for Optimization Purposes

Frozen stocks (−80 °C) of the strain *S. xylosus* CTC1642 (about 10^8^ CFU/mL) were quickly thawed and used to inoculate at different concentrations (CFU/mL) in 50 mL Falcon tubes filled with 10 mL of TSB broth. The actual viable cell counts were determined immediately after inoculation by plating decimal dilutions of samples onto TSA.

For phage propagation, cultures were infected with different concentrations (PFU/mL) of phage phiIPLA-RODI. The combined effect of initial phage titer, initial host concentration, temperature, and agitation on phage yield (final phage titer) and phage amplification ratio (estimated as the difference between final phage titer and the initial phage titer, expressed in log_10_ values) was evaluated after 3.5 h of incubation. The phage titer was determined as described in the previous section.

### 2.6. Experimental Design

The assays consisted of three successive designs that allowed estimating the effects of four independent continuous variables (initial phage titer, initial bacterial concentration, temperature, and agitation) on the phage yield and optimizing the conditions that could eventually result in the highest phage yield. The first design was used for an initial exploration of the experimental region in which phage production was expected to occur. It consisted of a quadratic Central Composite Design with the following ranges of the variables: initial phage titer (5.00 to 8.00 log_10_ PFU/mL); initial bacterial concentration (6.0 to 8.0 log_10_ CFU/mL); temperature (21 to 37 °C) and agitation (20 to 250 rpm). The objective of the second design was focused on the effect of temperature, using a D-optimal design, at fixed agitation (135 rpm). It had the following ranges for the variables: initial phage titer (6.0 to 8.0 log_10_ PFU/mL); initial bacterial concentration (5.0 to 7.0 log_10_ CFU/mL) and temperature (34 to 40 °C). Finally, the third design aimed at developing a Response Surface equation, using Central Composite Design, to predict phage production in the region of highest yield. It included only the initial phage titer (5.79 to 7.21 log_10_ PFU/mL) and initial bacterial concentration (5.59 to 8.41 log_10_ CFU/mL), while temperature and agitation were fixed at 38 °C and 135 rpm, respectively. The characteristics of these designs (and their respective yields) are summarized in Table 1. The levels of variables for all the designs were given by the program Design-Expert software version 7.0 (State-Ease, Inc., Minneapolis, MN, USA), provided their ranges and type of design. The order of the run performance was always randomly chosen. However, the values for initial bacterial populations, although intended to be those proposed by the designs, were difficult to fix accurately. Therefore, the actual bacterial concentrations reached just after inoculation, as determined by viable cell counts, were used for the statistical analysis.

### 2.7. Analysis of Results, Model Validation, and Final Response Surface (RS) Equation

The effects of the above-indicated variables (factors) on phage propagation (initial phage titer (A), initial bacterial concentration (B), temperature (C) and agitation (D)) were analyzed by the response surface methodology (RSM), using the following general quadratic model:

$$y=\beta_{0}+\sum_{i}^{k}\beta_{i}x_{i}+\sum_{i}^{k}x_{i}^{2}+\sum_{i}^{k}\sum_{j>i}^{k}\beta_{ij}x_{i}x_{j}$$

where *k* was 4, 3 and 2 for first, second, and third design, respectively [27]. The significant influential variables were those suggested by the sequential sum of squares (Type I) and supported by the corresponding ANOVA (partial sum of squares type III). The final models were obtained by a stepwise process, using *p* ≤ 0.05 and *p* ≥ 0.10 as criteria for entering and removal of variables, respectively. The effects of the variables and model fits were also checked graphically. For the validation of the third model, nine additional assays were performed with the initial phage titer fixed at 6.5 log_10_ PFU/mL and the initial bacterial populations around the levels of maximal phage yield. A final equation for the model was developed by enlarging the data from the third design with the validation results. This last model was used for obtaining the conditions which maximize the phage yield and amplification.

### 2.8. Statistical Analysis

Statistical analyses for phage propagation were performed using the statistical package IBM SPSS Statistics for Windows Version 23 (IBM Corp. Armonk, NY, USA). Data related to phage propagation carried out with the control host (*S. aureus* IPLA1), and test host (*S. xylosus* CTC1642) were subjected to one-way ANOVA, and the Student-Newman-Keuls (SNK) test was used for comparison of means at a level of significance *p* < 0.05. Three biological replicates were used in all the assays.

For optimizing the phage propagation on *S. xylosus* CTC1642, the experiments were always designed and analyzed using Design-Expert software version 7.0 (State-Ease, Inc., Minneapolis, MN, USA). Final optimization was achieved using the desirability approach, which finds operating conditions that provide the “most desirable” response values. The desirability function d_i_ (Y_i_) assigns numbers between 0 (undesirable value) and 1 (ideal response) to the possible values of Y_i_ (phage yield or phage amplification ratio). Usually, the individual desirability values are combined using the geometric mean, which gives the overall desirability (D) which is maximized with respect to the controlled variables.

## 3. Results

### 3.1. PhiIPLA-RODI Infects Food-Grade S. xylosus Strains and Other Staphylococcal Species

To avoid amplification of virulence genes or the risk of accidental contamination with the original host (*S. aureus*), the ability of phage phiIPLA-RODI to infect food-grade staphylococcal strains was tested. The sensitivity of three *S. xylosus* and four *S. carnosus* strains was determined by the plaque assay, and the efficiency of plating (EOP) was also calculated. The EOP was determined by comparison to the reference strain *S. aureus* IPLA1. None of the *S. carnosus* strains and *S. xylosus* CTC1638 was sensitive to the phage. By contrast, EOP values of 0.31 and 0.00004 were obtained for *S. xylosus* CTC1642 and *S. xylosus* CTC1644, respectively. According to these results, *S. xylosus* CTC1642 was selected as a potential host for further phage propagation.

**Figure 1:**
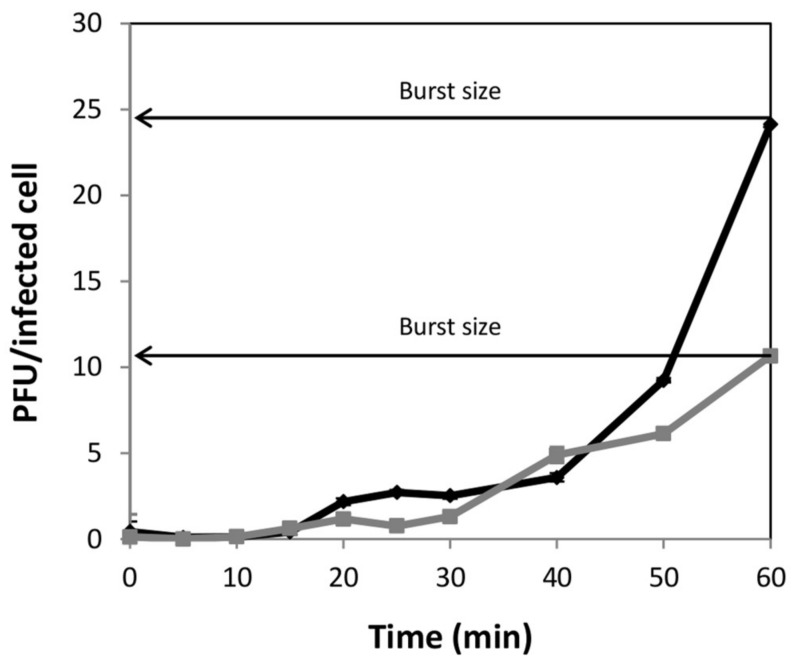
One-step growth curves of phiIPLA-RODI on *S. aureus* IPLA1 (black diamonds) and *S. xylosus* CTC1642 (grey squares), respectively. Values correspond to the number of plaque-forming unit (PFU) per infected cell. Each data point shows the mean ± standard deviation for three independent experiments.

One-step growth curves of phage phiIPLA-RODI, pre-amplified on *S. aureus* IPLA1, were obtained on both *S. aureus* IPLA1 and *S. xylosus* CTC1642 (Figure 1). Values of burst size (number of viral particles per infected cell) determined at 60 min after infection were 25 and 10, respectively.

Data of phage phiIPLA-RODI propagation on *S. aureus* IPLA1 were compared with those obtained on *S. xylosus* CTC1642 under the same experimental conditions used in conventional phage propagation, as explained in M&M section. The titer of lysates obtained using *S. aureus* IPLA1 as a host strain (8.9 ± 0.1 log_10_ PFU/mL) was significantly higher (*p <* 0.05) than the value of suspensions propagated on *S. xylosus* (8.2 ± 0.2 log_10_ PFU/mL).

Regardless of the staphylococcal species used for phage propagation, phiIPLA-RODI showed the same host range and similar EOPs on the different *Staphylococcus* species tested (Table S1). In addition, the phage propagation on IPLA1 and CTC1642 strains did not result in any significant differences in its biofilm removal ability (Figure S1).

### 3.2. Identification of Experimental Factors Affecting Phage Yield

The first experimental design consisted of 21 propagation runs which included the four experimental factors (A: initial phage titer, B: initial bacterial concentration, C: temperature and D: agitation) expected to influence the phage yield (Table 1). The levels used were those proposed by the software (except for bacteria which concentrations were those effectively reached in the experiment as commented above). The phage yield responses were analyzed by the sequential model sum of squares and the fit subjected to the corresponding ANOVA. From the analysis (Table 2), it was deduced that the effect of agitation on the phage yield was not significant (*p* > 0.05) in the range of 20–250 rpm (i.e., it was not retained). On the contrary, the initial phage titer and temperature showed significant (at *p* < 0.05) linear effects while the initial bacterial concentration had a significant (at *p* ≤ 0.10) quadratic effect. The retention of the non-significant linear term of the initial bacterial population is due to the application of the hierarchical principle, which establishes the maintenance of lower order term (linear in this case) when one of higher order is retained (Table 2).

**Figure 2:**
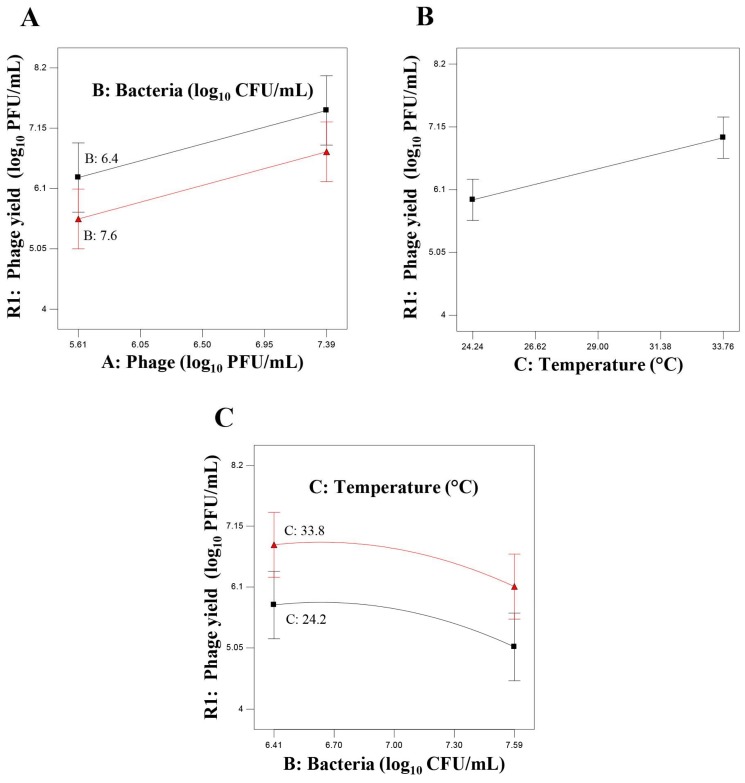
Plot of the first design response. Phage yield (response) as a function of: initial phage titer at fixed temperature (33.76 °C) and agitation (135 rpm) for two levels of bacteria (**A**); temperature at fixed initial phage titer (6.74 log_10_ PFU/mL), initial bacterial concentration (7.00 log_10_ CFU/mL) and agitation (135 rpm) (**B**); and initial bacterial concentration at fixed initial phage titer (6.43 log_10_ PFU/mL) and agitation (135 rpm) for two temperature levels (**C**).

These effects are shown graphically in Figure 2 as functions of several variables. Regardless of the initial bacterial population, an increase in the initial phage titer, within the ranges studied in this case, always led to a higher phage yield (Figure 2A). Also, a linear increase of the phage yield was observed as the temperature rose (Figure 2B). Moreover, as the initial population of bacteria and temperature increased, there were a progressive (quadratic) decrease and a linear increment of phage yield, respectively (Figure 2B,C).

Therefore, a new design was performed in which the agitation was fixed at an intermediate level (135 rpm) since its effect on phage yield was not significant (*p* > 0.05). On the contrary, the temperature was increased to the range 34–40 °C, due to the favorable linear effects previously observed (Figure 2B). Finally, the ranges of the initial phage and bacterial concentration were fixed from 6.00 to 8.00 log_10_ PFU/mL and from 5.00 to 7.00 log_10_ CFU/mL respectively, to include the experimental regions of high phage yield. 

**Figure 3:**
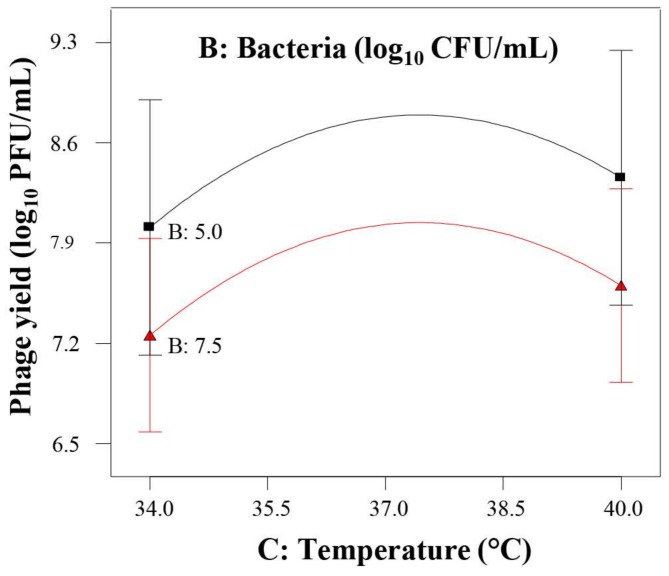
Plot of the second design response. Phage yield (response) as a function of temperature, at two initial bacterial concentrations, and fixed initial phage titer (7.32 log_10_ PFU/mL).

In these conditions, the effect of the initial phage concentration was not significant but the temperature had a significant quadratic effect (Table 3), regardless of the concentration of the initial bacterial population, and showed a clear optimum around 38 °C (Figure 3). As in the first design, the effect of the initial bacterial population on the phage yield was also quadratic and significant at *p* ≤ 0.10 (Table 3) with a high initial bacterial population resulting in a lower phage yield (Figure 3), regardless of temperature. Hence, the most relevant conclusion of this design was the quadratic effect of temperature and the identification of the level for maximum phage yield.

### 3.3. Response Surface Model for Phage phiIPLA-RODI Yield

The results from the previous design led to planning a third one in which temperature was fixed at its optimum (38 °C) and agitation at 135 rpm, while the ranges of phage and bacteria were maintained similar (Table 1, third design). However, as commented above, the design levels for the theoretical initial bacterial concentrations were difficult to reach and resulted in the actual values included in Table 1, which were used for the statistical analysis. The analysis of this design revealed, within the ranges of the variables assayed, a non-significant effect of initial phage titer and a persistent quadratic significant effect of the initial bacterial population (Table 4). The equation had the following expression:

(1) Phage yield (log10PFUmL)=−60.36+20.46×bacteria−1.50×bacteria2

**Figure 4:**
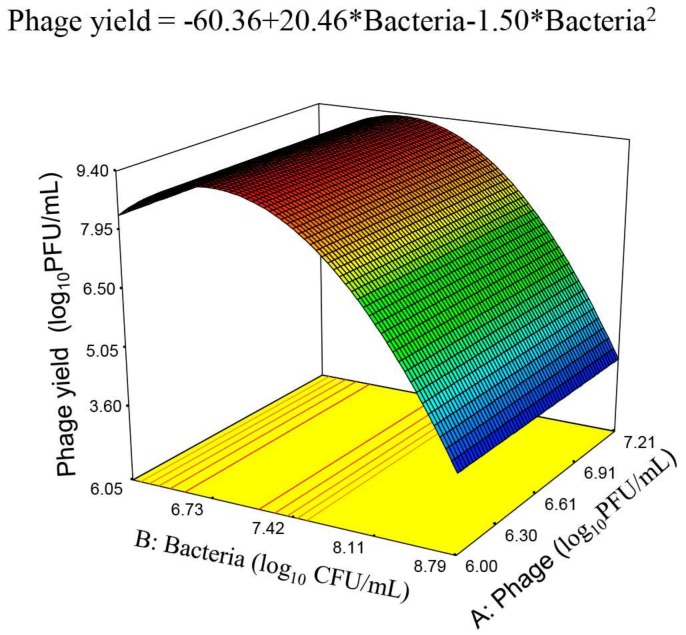
Response surface plot of phage yield as a function of the initial bacterial population and phage titer, based on the experiments from the third design, with a maximum at 6.82 log_10_ CFU/mL initial bacteria.

The model showed a significant fit and an insignificant lack of fit (Table 4). The response surface plot of the equation (Figure 4) was a plane with a slightly rising hill which reached its maximum at an initial bacterial concentration of 6.82 CFU/mL. Then, it decreased sharply at higher bacterial concentrations because of the negative sign and quadratic exponent of this variable (Equation (1)). Moreover, the phage titer has been particularly high in some experiments with up to 9.3 log_10_ PFU/mL (Table 1), the highest titer found so far. That is, the third design pointed to combinations of the variables which resulted in maximum response (Figure 4), due to the Equation (1) structure.

### 3.4. Validation of RSM

Once reached the region of maximum phage production, nine validation experiments were performed at the levels specified in Table 5. There were no differences between the experimentally observed and predicted values. Therefore, the model (Equation (1)) could be used to make predictions.

### 3.5. Final Equations for the Phage Production and Phage Amplification Ratio

The results from the third experimental design were enlarged with those obtained in the validation tests. The new data set was then used to estimate a definitive RS from a higher number of responses and degrees of freedom. The model estimated was, as in the case of the third design, a quadratic function of the initial bacterial concentration which also retained the linear term to preserve the hierarchical principle (Table 6). Its equation, expressed in terms of the physical variable units, was the following:

(2) Phage yield (log10PFUmL)=−63.38+21.24×bacteria−1.55×bacteria2

where bacteria is the initial bacterial concentration in the culture medium, expressed in log_10_ CFU/mL. The Equation (2) was quite similar to that obtained when using only the data from the third design (Equation (1)) and also reached the maximum at a very close initial bacterial concentration (6.85 vs. 6.82 log_10_ CFU/mL). The predicted phage yields for the validation data using this final equation were similar to those deduced previously from the third design, but the predictions had a lower dispersion (0.5 vs. 0.3 SE) (Table 5, last two columns).

After optimization, propagation of phiIPLA-RODI on *S. xylosus* CTC1642 reached an average titer of 8.9 ± 0.1 log_10_ PFU/mL, which was not significantly different from that obtained with the reference strain of *S. aureus* IPLA1 (8.9 ± 0.2 log_10_ PFU/mL) (*p* > 0.05).

However, not only the phage yield is important but also the phage amplification ratio (i.e., the times the phage population (expressed in log_10_) is multiplied during the propagation process). The RS model estimated was also significant, had a non-significant lack of fit (Equation (3)), and was quite similar to that previously deduced for phage yield (Equation (2)). It took the following form:

(3) $$Phage\;amplification\;ratio=-60.58+18.63\times bacteria-1.37\times bacteria^{2}$$

**Figure 5:**
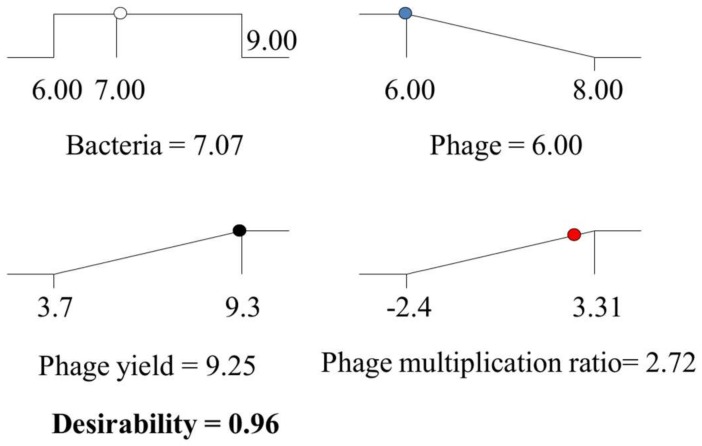
Optimization of both final phage yield and phage multiplication ratio. Final phage yield (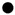) and phage multiplication ratio (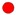), as a function of initial bacterial populations (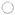) and phage titers (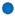), using the desirability approach as implemented in Design Expert.

Mathematically, regardless of the initial phage population, the optimum phage amplification ratio (or population multiplication) is also situated at bacterial concentrations around 6.8 log_10_ CFU/mL (18.63/2 × 1.37, since the first derivative at the maximum, should be null). However, it may also be obtained considering the initial bacteria and phage titer simultaneously. With this objective, the desirability approach was applied, using the following criteria: initial bacterial concentration within the range 6–9 log_10_ CFU/mL, minimum initial phage titer in the range 6–8 log_10_ PFU/mL, maximum phage yield and phage amplification ratio. The results indicated that, by using initial bacterial and phage concentrations of about 7.07 log_10_ CFU/mL and 6.00 log_10_ PFU/mL, respectively, a remarkable phage yield (9.25 ± 0.9 log_10_ PFU/mL) and phage amplification ratio (2.72 ± 0.90) could be obtained (Figure 5), with a total desirability of 0.96 (quite close to the ideal value of 1.00). Therefore, the process may reach the highest phage yield and, at the same time, maximum amplification (the initial phage is increased by almost 3 log units).

## 4. Discussion

To exploit the advantages of bacteriophages as antimicrobials, it is essential to adapt their production to meet quality and safety requirements for sustainable phage therapy products [28]. In this context, the use of a surrogate host such as a food-grade bacterium minimizes the risk of toxin or host contamination on phage preparations. The ability of phage phiIPLA-RODI to infect a wide spectrum of staphylococcal strains [16] allowed selecting a *S. xylosus* strain, from meat origin, to propagate the phage. A reduction in the phage yield (final phage titer) could be expected due to the lower burst size calculated in *S. xylosus* compared with *S. aureus* (10 versus 25 phage particles per infected cell). In this regard, a reduction in the burst size was also observed when the *Salmonella* phage phi PVP-SE3 was propagated in the non-pathogenic strain *E. coli* BL21 [29], while a similar number of phage particles per infected cell were obtained after infection of *S. xylosus* by the *S. aureus* infecting phage Team1 [30]. As previously observed in other phages [30], the host range and the EOP value shown by phiIPLA-RODI when propagated on both *S. xylosus* CTC1642 and *S. aureus* IPLA1 were fairly similar. Besides, the ability of phiIPLA-RODI to remove biofilms was similar regardless of the bacterial host used for phage propagation. Overall, the lack of pathogenicity of the host strain *S. xylosus* CTC1642 and consequently, the safety of the final phage product justify, in our opinion, the use of the food-grade strain even though a lower phage yield is obtained. In an attempt to counteract this disadvantage, we optimized the phiIPLA-RODI propagation process by using RSM since it is handy for studying processes in which the response is influenced by several variables [22]. In fact, this method was previously used to optimize the production of an *E. coli* infecting filamentous phage [23]. It should be noticed, however, that filamentous phages do not reproduce by lysing bacteria, instead, they are secreted into the environment without killing the host, while the life cycle of myovirus phages ends with the lysis of the host. Likewise, Grieco et al. [23] have used temperature, dissolved oxygen and pH as independent variables while, in our study, phage and bacterial concentration, temperature and agitation were tentatively considered initial influential on the phage yield when *S. xylosus* is used as propagation host. Remarkably, the initial bacterial concentration was particularly significant for phiIPLA-RODI propagation as it had a quadratic effect on the phage yield.

The propagation process is the result of a succession of several infection cycles, and each of them consists of an adsorption period, nucleic acid uptake, latent period and progeny release. The duration of these steps and the number of phage particles produced per infected cell will determine the phage yield [31,32]. It is well known that phage-host interactions are affected by environmental conditions that alter the physiological state of bacteria, through changing their susceptibility to phage infection and the phage productivity [33]. Moreover, optimal bacteriophage infection traditionally requires bacterial host growing exponentially, although some bacteriophages like T4 can efficiently infect *E. coli* in stationary phase and kill the host after a hibernation period [34].

In this context, a first design was used as initial guidance for approaching the production of phages in an experimental region (space limited by the range of variables) in which, according to previous non-systematic assays, high phage yield (response) were expected. The results showed that agitation within the range assayed did not affect phage yield, however, agitation might favor the encounter between bacteria and phage needed for the phage infection to take place and prevent the bacterial cell from sedimentation. Therefore, the use of an intermediate level (135 rpm) was fixed for further assays. For the variable temperature, a wide range of 21–37 °C was initially selected to include values even lower than the optimal for *Staphylococcus* growth because other authors have previously observed improved phage production at temperatures below the optimum for bacterial host growth [23]. The data from the assays proposed by the first design pointed out that the range of temperature was not appropriate since the effect on the phage yield was linear and increased progressively as the temperature rose. Therefore, these results supported the notion of using a higher temperature around those that favor host metabolism for additional designs. The linear effect of initial phage titer and the quadratic effect of the initial bacterial concentration on the phage yield also suggested to increase the initial phage concentration and to decrease the initial bacteria concentration. Hence, the analysis of the data from the experiments proposed by the first design reduced the influential variables to three: initial phage titer, initial bacterial concentration and temperature.

Subsequently, the main goal of the second design was exploring the effect of temperature in detail, covering the region of optimal host growth and, eventually, the optimum level of phage yield. Its results confirmed the pertinence of increasing temperature and showed a quadratic effect defining the optimum temperature at 38 °C that was adopted for further assays. The range of the initial bacterial population was still appropriate, and the ANOVA led to the identification of a quadratic effect (*p* ≤ 0.10) for this variable, in agreement with the results of the first design. The effect of initial phage concentration was not statistically significant under the experimental conditions proposed by the second design. The reason for this observation could be the different ranges of temperature tested. Indeed, it is well known that the lytic phage infection process is very much dependent on the bacterial host metabolic machinery. Therefore, a temperature that improves bacterial metabolic activity will result in a better phage yield. In this regard, it should be noted that the range used in the second design (34–40 °C) is more appropriate for the bacterial host growth than that used in the first design (21–37 °C), resulting in an enhanced phage yield and consequently, the initial phage concentration has not longer statistically significant effect on the response.

Once the optimum temperature was defined, the influential variables were reduced to only two: initial phage titer and bacterial concentration. Further analysis of the relationship between these variables was approached by the third design in which the interest was focused on finding the equation of the RS defining the region of maximum phage production, already outlined from the results of the previous design. The ranges of both variables were established taking into account that, for an efficient infection process, phages should be able to encounter the bacterial cell host. For this purpose, the bacterial population must exceed its proliferation threshold [35,36], to facilitate the initial phage titer to increase. It should be noticed that MOI (as phage/bacteria ratio) has not been used as independent variable in the current study because similar MOI values are obtained from different concentrations of initial phage and bacteria. In fact, the particular phage and bacteria concentrations used for phage propagation did really result in different values of the final phage titer. Having fixed agitation and temperature, the data from the experimental assays of the third design confirmed the quadratic effect of bacteria regardless of the initial phage titer, which was not significant within the studied range. All the parameters related to the model fit were appropriate. In addition, several independent validation experiments showed also good agreement with its predictions and, remarkably, the incorporation of validation data into those from the third model allowed deducing a final RS model with a higher degree of freedom for the medium standard error (MSE) and narrower confidence limits for the estimations. Also, from the third model, enlarged with the validation data, a final model could be deduced which led not only to the conditions of optimal phage production but also revealed a sharp decrease in the phage yield at bacterial concentrations above 7.5 log_10_ CFU/mL. This behavior could be due to the adsorption of a proportion of the new viral progeny to the host cells that were not initially infected, which could partially hamper its detection by phage titration.

The optimization process allowed therefore maximizing the phage yield and the phage amplification ratio, while minimizing the initial phage concentration and using an initial bacterial population between the ranges studied in the third design. The optimization process deduced several possible combinations. Among them, it was chosen that providing the highest desirability which consisted of the initial bacterial population, 7.07 log_10_ CFU/mL; and initial phage titer, 6.00 log_10_ PFU/mL (Figure 5), while maintaining agitation (135 rpm) and temperature (38 °C) at their fixed levels. Using these conditions, it is expected a phage yield of up to 9.25 ± 0.35 log_10_ CFU/mL along with a phage amplification ratio of 2.72 log units over the initial phage titer. The global desirability, defined as the geometric mean of desirability values of the phage yield and phage amplification ratio, would be in this case high (0.96) and fairly close to the most “desirable” response 1.00. These results support the use of the food-grade strain *S. xylosus* CTC1642 as an appropriate alternative of the pathogenic strain *S. aureus* IPLA1 for phiIPLA-RODI propagation at the setting conditions established by the final RS model. The setting parameters could be the starting point for performing the upscaled production of the phage that would be required for its potential use in clinical [28] and food safety [15] applications.

## Figures and Tables

**Table 1:** Experimental designs used for optimizing the phage yield (response) as a function of temperature, initial bacterial concentration, initial phage titer and agitation. Responses are also included.

	1st Tentative Design (Central Composite)					2nd Design (D-Optimal) ^a^				3rd Design (Central Composite) ^b^		
Runs	Phage Titer	Bacterial Concentration	Temperature (°C)	Agitation (rpm)	Phage Yield	Phage Titer	Bacterial Concentration	Temperature (°C)	Phage Yield	Phage Titer	Bacterial Concentration	Phage Yield
1	7.39	7.56	24.2	67	5.53	8.00	5.78	40.0	7.42	7.00	6.85	8.4
2	6.50	7.28	29.0	135	5.67	6.00	5.95	34.0	6.61	6.50	7.72	9.0
3	6.50	8.27	29.0	135	4.37	7.19	7.45	37.6	8.43	6.50	7.35	9.3
4	5.61	6.96	33.8	67.	6.66	7.19	6.30	34.0	6.86	6.50	6.05	8.5
5	5.00	7.53	29.0	135	4.86	6.00	7.44	34.0	7.57	7.21	7.61	8.9
6	5.61	7.99	33.8	203	4.08	7.18	6.48	37.8	7.37	5.79	7.41	9.1
7	6.50	7.51	29.0	135	5.79	8.00	6.81	40.0	7.81	6.00	8.15	3.7
8	6.50	7.62	29.0	135	6.00	8.00	5.66	36.1	8.27	7.00	8.11	8.3
9	6.50	7.38	21.0	135	5.51	6.00	6.66	37.6	8.05	6.50	7.61	9.0
10	6.50	6.25	29.0	135	6.13	6.00	7.82	40.0	8.12	6.50	7.34	9.0
11	6.50	7.57	29.0	135	5.68	6.00	7.43	40.0	8.27	6.00	6.29	8.7
12	8.00	7.11	29.0	135	6.80	8.00	7.48	34.0	8.04	6.50	7.53	9.1
13	5.61	7.88	24.2	203	4.23	6.00	5.77	34.0	7.58	6.50	8.40	4.1
14	7.39	7.01	24.2	203	7.06	6.70	5.86	40.0	7.54			
15	5.61	6.99	24.2	67	5.25							
16	6.50	7.04	29.0	20	5.02							
17	6.50	7.11	29.0	135	5.26							
18	6.50	6.85	37.0	135	8.13							
19	7.39	7.15	33.8	203	7.34							
20	7.39	7.70	33.8	67	6.83							
21	6.50	7.18	29.0	250	6.43							

Note: Phage titer and phage yield are expressed as log_10_ plaque-forming unit (PFU)/mL and bacterial concentration is expressed as log_10_ colony-forming unit (CFU)/mL. The bacterial concentrations correspond to the effective levels reached in the experiment. Standard deviations for the design values (as estimated from the analysis of variance (ANOVA) pure error) were 0.34, 0.14, 0.13 log_10_ PFU/mL, respectively. ^a,b^ Agitation fixed at 135 rpm. ^b^ Temperature fixed at 38 °C.

**Table 2:** ANOVA for Response Surface Reduced Quadratic Model (partial sum of squares type III) of the first design.

Source	Sum of Squares	Degrees of Freedom	Mean Square	*F* Value	*p*-Value (Prob > *F*)
**Model**	16.99	4	4.25	12.45	<0.0001 significant
**A-*Phage***	4.46	1	4.46	13.09	0.0023
**B-*Bacteria***	0.78	1	0.78	2.28	0.1508
**C-*Temperature***	3.62	1	3.62	10.62	0.0049
**B^2^**	1.21	1	1.21	3.56	0.0775
**Residual**	5.46	16	0.34		
**Cor total**	22.44	20			

Notes: The term B-*Bacteria*, was introduced in application of the hierarchical principle. Alfa to enter, *p* ≤ 0.05, alfa to exit, *p* ≥ 0.10.

**Table 3:** ANOVA for Response Surface Reduced Quadratic Model (partial sum of squares type III) of the second design.

Source	Sum of Squares	Degrees of Freedom	Mean Square	*F* Value	*p*-Value Prob > *F*
**Model**	2.51	4	0.63	4.59	0.0271
**B-*Bacteria***	0.13	1	0.13	0.91	0.3644
**C-*Temperature***	0.30	1	0.30	2.19	0.1734
**B^2^**	0.52	1	0.52	3.76	0.0844
**C^2^**	0.86	1	0.86	6.28	0.0336
**Residual**	1.23	9	0.14		
**Cor total**	3.75	13			

Notes: The term B-*Bacteria* and C-*Temperature* were introduced in application of the hierarchical principle. Alfa to enter, *p* ≤ 0.05, alfa to exit, *p* ≥ 0.10.

**Table 4:** ANOVA for Response Surface Reduced Quadratic Model (partial sum of squares type III) of the third design.

Source	Sum of Squares	Degrees of Freedom	Mean Square	*F* Value	*p*-Value Prob > *F*
**Model**	37.26	2	18.63	20.01	<0.0001 significant
**B-*Bacteria***	2.15	1	2.15	2.30	0.1455
**B^2^**	18.16	1	18.16	19.50	0.0003
**Residual**	1.04	18	0.058		
**Lack of fit**	0.78	16	0.049	0.37	0.9042 not significant
**Pure error**	0.26	2	0.13		
**Cor total**	4.03	19			

Notes: The term B-*Bacteria* was introduced in application of the hierarchical principle. Alfa to enter, *p* ≤ 0.05, alfa to exit, *p* ≥ 0.10.

**Table 5:** Experimental conditions, predicted responses (±SE) according to the RS model developed for the 3rd experimental design, and actual results for the validation experiments.

Initial Bacteria Population	Initial Phage Titer	Phage Yield, Validation Experiments	Predicted Phage Yield, RS 3rd Design	Predicted Phage Yield, RS Enlarged 3rd Design ^a^
7.51	6.50	8.8 ± 0.1	8.6 ± 0.5	8.7 ± 0.3
7.42	6.50	8.8 ± 0.1	8.7 ± 0.5	8.8 ± 0.3
7.63	6.50	8.8 ± 0.1	8.3 ± 0.5	8.4 ± 0.3
7.56	6.50	8.7 ± 0.1	8.5 ± 0.5	8.5 ± 0.3
7.72	6.50	8.8 ± 0.1	8.1 ± 0.5	8.2 ± 0.3
7.62	6.50	8.8 ± 0.1	8.3 ± 0.5	8.4 ± 0.3
7.35	6.50	8.6 ± 0.1	8.9 ± 0.5	8.9 ± 0.3
7.28	6.50	8.5 ± 0.1	9.0 ± 0.5	9.0 ± 0.3
7.39	6.50	8.8 ± 0.1	8.8 ± 0.5	8.9 ± 0.3

Note: bacteria and phage populations are expressed as log_10_ CFU/mL and 1og_10_PFU/mL, respectively. ^a^ Predicted responses (±SE) based on the final Response Surface including the validation data.

**Table 6:** ANOVA for Response Surface Reduced Quadratic Model (partial sum of squares type III) based on the data from the third design and the validation data.

Source	Sum of Squares	Degrees of Freedom	Mean Square	*F* Value	*p*-Value Prob > *F*
**Model**	37.26	2	18.63	20.01	<0.0001 significant
**B-*Bacteria***	2.15	1	2.15	2.30	0.1455
**B^2^**	18.16	1	18.16	19.50	0.0003
**Residual**	1.04	18	0.058		
**Lack of fit**	0.78	16	0.049	0.37	0.9042 not significant
**Pure error**	0.26	2	0.13		
**Cor total**	4.03	19			

Notes: The term B-*Bacteria* was introduced in application of the hierarchical principle. Alfa to enter, *p* ≤ 0.05, alfa to exit, *p* ≥ 0.10.

## Supplementary Materials

Supplementary materials can be found at http://www.mdpi.com/1999-4915/10/4/153/s1.
