# Supplementary material for: Optimizing Propagation of Staphylococcus aureus Infecting Bacteriophage vB_SauM-phiIPLA-RODI on Staphylococcus xylosus Using Response Surface Methodology
Source: Viruses. 2018 Mar 27;10(4):153. doi: 10.3390/v10040153 (PMC5923447; doi:10.3390/v10040153)
Supplement: Supplementary file 1 [file viruses-10-00153-s001.zip › Supplementary material/Figure S1.docx]

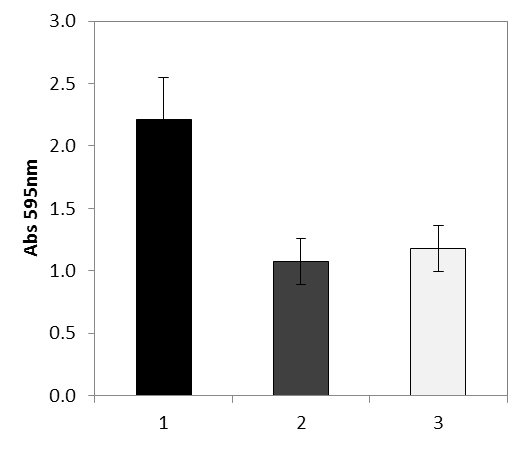


a

b

b

**Figure S1.** Bacteriophage mediated removal of 24 h-old biofilms of S. aureus IPLA16. Biofilms were treated with phage phiIPLA-RODI propagated on *S. aureus* IPLA1 (dark grey) and on *S. xylosus* CTC1642 (light grey). Control biofilms are shown in black. Biomass was calculated by crystal violet staining of adhered cells after phage treatment (test samples) and without phage treatment (control samples). Absorbance was measured at a wavelength of 595 nm. Means and standard deviations were calculated from three biological replicates. Bars having different letters are significantly different (ANOVA; P<0.05).
