# Supplementary material for: Optimizing Propagation of Staphylococcus aureus Infecting Bacteriophage vB_SauM-phiIPLA-RODI on Staphylococcus xylosus Using Response Surface Methodology
Source: Viruses. 2018 Mar 27;10(4):153. doi: 10.3390/v10040153 (PMC5923447; doi:10.3390/v10040153)
Supplement: Supplementary file 1 [file viruses-10-00153-s001.zip › Supplementary material/Table S1.docx]

**Table S1.** Efficiencies of plaque formation (EOP) of phage phiIPLA-RODI propagated on *S. aureus* IPLA1 (A) and *S. xylosus* CTC1642 (B), on different sensitive strains.

| **Specie** | **Strain** | **Origin** | **References** | **EOP^a^** | |
| --- | --- | --- | --- | --- | --- |
|  |  |  |  | **A** | **B** |
| *S. aureus* | IPLA1^b^ | Dairy industry surfaces | 24 | 1.00 ± 0.10 | 1.00 ± 0.20 |
|  | IPLA15 | Meat industry surfaces | 24 | 0.29 ± 0.18 | 0.31 ± 0.10 |
|  | IPLA16 | Meat industry surfaces | 24 | 1.33 ± 0.29 | 0.99 ± 0.4 |
| *S. lugdunensis* | ZL5-11 | Women’s breast milk | 16 | 1.02 ± 0.35 | 1.30 ± 0.20 |
| *S. pasteuri* | ZL16-6 | Women’s breast milk | 16 | 0.33 ± 0.05 | 0.53 ± 0.22 |
| *S. arlettae* | ZL114-5 | Women’s breast milk | 16 | 0.37 ± 0.06 | 0.54 ± 0.06 |
| *S. xylosus* | ZL61-2 | Women’s breast milk | 16 | 0.09 ± 0.04 | 0.10 ± 0.03 |
| *S. gallinarum* | ZL90-5 | Women’s breast milk | 16 | 0.38 ± 0.09 | 0.27 ± 0.05 |
| *S. kloosii* | ZL74-2 | Women’s breast milk | 16 | 0.50 ± 0.18 | 0.30 ± 0.02 |
| ^a^ Means and standard deviations were calculated from three biological replicates.  ^b^ The reference strain used for the EOP calculations. | | | | | |
